# Efficacy of mindfulness and goal setting interventions for increasing resilience and reducing smoking in lower socio-economic groups: randomised controlled trial protocol

**DOI:** 10.1186/s13722-022-00355-w

**Published:** 2023-02-06

**Authors:** Reece De Zylva, Elissa Mortimer, Emma Miller, George Tsourtos, Sharon Lawn, Carlene Wilson, Jonathan Karnon, Richard Woodman, Paul Ward

**Affiliations:** 1 Research Centre for Public Health, Equity and Human Flourishing, Torrens University Australia, 88 Wakefield St, Adelaide, SA 8000 Australia; 2 College of Medicine and Public Health, Flinders University, Bedford Park, SA Australia; 3 The Stretton Institute, The University of Adelaide, Adelaide, SA Australia; 4 School of Psychology and Public Health, La Trobe University, Bundoora, VIC Australia; 5 Olivia Newton-John Cancer Wellness and Research Centre, Austin Health, Heidelberg, VIC Australia

**Keywords:** Smoking cessation, Socioeconomic status, RCT, Resilience, Mindfulness, Goal setting, Cognitive behavioural therapy, MiCBT, Peer support, Cost effectiveness

## Abstract

**Background:**

Smoking and resulting health problems disproportionately impact low socioeconomic status (SES) individuals. Building resilience presents an approach to ‘closing the gap’. Mindfulness-based interventions and setting realistic goals are preferred in low socioeconomic communities. We aim to test if these interventions, delivered online and consolidated with peer support offered via ex-smokers, are successful in promoting smoking cessation and resilience. Our conceptualisation of resilience encompasses the inner capacity/skills and external resources (e.g., social support) which smokers utilise to bounce back from adversity. We include a process evaluation of barriers/facilitators to interventions and cost-effectiveness analysis (from health system perspective).

**Methods:**

We plan a four-arm parallel 12-month RCT with a 6-month follow-up to test the efficacy of three group-based interventions each followed by peer support. Arm 1: mindfulness-integrated cognitive behavioural therapy; Arm 2: mindfulness training; Arm 3: setting realistic goals; Arm 4: active control group directed to quit services. All interventions will be administered online. Participants are adult smokers in Australia (N = 812) who have an average weekly household income less than $457AUD or receive welfare benefits. Group-based interventions will occur over 6 months, followed by 6 months of forum-based peer support. Primary outcome: self-reported 14-day period prevalence of smoking abstinence at 6 months, with remote biochemical verification of saliva cotinine (< 30 ng/mL). Secondary outcomes include: internal resilience (Connor-Davidson Resilience Scale-25); external resilience (ENRICHD social support tool); quality adjusted life years (EQ-5D-5L); self-efficacy for smoking abstinence (Smoking-Abstinence Self-Efficacy Questionnaire); motivation to quit smoking (Biener and Abrams Contemplation Ladder); nicotine dependence (Fagerstrom Test for Nicotine Dependency); equanimity (Equanimity Scale-16); stress (Perceived Stress Scale-10); goal assessment/attainment (Problems and Goals Assessment Scale).

**Discussion:**

This study is the first to compare resilience interventions for low SES smokers which have been identified by them as acceptable. Our various repeated measures and process evaluation will facilitate exploration of mechanisms of impact. We intervene within the novel framework of the Psychosocial Model of Resilience, applying a promising paradigm to address a critical and inequitable public health problem.

*Trial registration* Australian New Zealand Clinical Trials Registry ID: ACTRN12621000445875, registered 19 April 2021 (https://anzctr.org.au/Trial/Registration/TrialReview.aspx?id=381007&isReview=true). The Universal Trial Number is U1111-1261–8951

**Supplementary Information:**

The online version contains supplementary material available at 10.1186/s13722-022-00355-w.

## Background

Smoking tobacco is a leading cause of preventable death globally, resulting in approximately eight million deaths each year when including the impact of second-hand smoke. [1] Diseases associated with smoking—such as lung cancer, heart disease, and stroke—are, if not fatal, detrimental to quality of life. In Australia, for instance, an estimated six million quality adjusted life years (QALYs) will be lost from smoking if the current population of smokers is tracked until 70 years of age. [2] Globally, the economic burden on healthcare systems and lost productivity from smoking-attributable diseases in 2012 was estimated at purchasing power parity of $1852 billion for the year, or 1.8% of the world’s annual gross domestic product. [3]

Although population prevalence of smoking is generally decreasing in high-income countries, [1] those in socioeconomically disadvantaged groups are less successful at quitting and are over-represented in smoking statistics. [4] Socioeconomic status (SES) is a broad concept which refers to the position of an individual, family, or group within the socioeconomic hierarchy, and as it relates to health, the consequential environment and access to basic resources. [5] It is typically measured by one or several of the following factors: income/wealth, education, and occupation—though in practice, the categorisation of SES will vary between countries and over time. Individuals from lower SES environments are more likely to try smoking, smoke regularly, [4] and die prematurely from smoking-attributable diseases. [6] Furthermore, smokers in this group are likely to have a higher nicotine dependence. [7, 8] Although low SES individuals are likely to make quit attempts at a similar rate to other socio-economic strata, they are less likely to succeed. Kotz and West [9] found that the success rate of quit attempts among those in the highest socioeconomic level was 20.4%, compared to 11.4% for those in the lowest socioeconomic level, but found no socioeconomic-related differences in the rate of quit attempts.

**Fig. 1:**
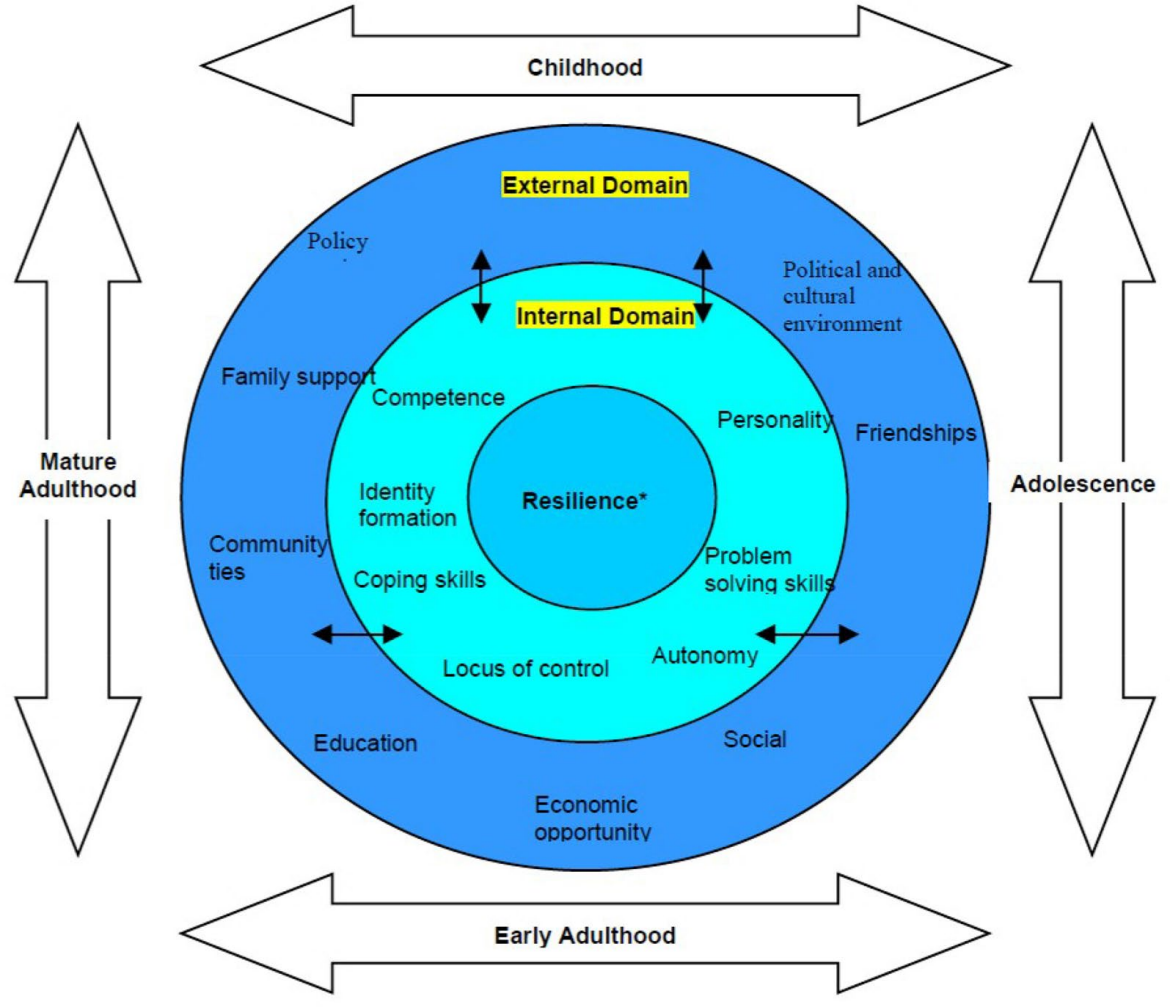
The psychosocial interactive model of resilience [17]

Building resilience could be vital for promoting smoking cessation in low SES groups. This asset-based approach focuses on building strengths, capabilities, and protective factors, as opposed to fixating on deficits of people or groups. [10–15] Resilience is defined not merely as coping with perceived difficulties but “bouncing back from adversity” and finding hope and meaning. [16] Our conceptual model of resilience (Fig. 1; [15, 17]) extends this notion beyond the internal properties of the individual (internal resilience) and into the social environment and interpersonal resources (external resilience). Internal factors such as self-efficacy, self-confidence, and motivation are often important for building resilience and hence making successful quit attempts. However, individuals with a lower SES, on average, have significantly lower levels of motivation and self-efficacy compared to higher SES individuals. [4, 8, 18–22] These factors are correlated with feelings of powerlessness, less social support, higher perceived stress eroding motivation, and normalisation of smoking behaviour. [8, 23] Given these multifarious obstacles to cessation faced by low SES individuals and the proposed interplay between internal and external resources (Fig. 1), building resilience presents as a promising approach from which to empower low SES individuals to change their smoking behaviour.

The Behaviour Change Wheel [24] is the guiding framework for resilience interventions for smoking cessation in the present study. It synergises with the Psychosocial Model of Resilience because it describes a comprehensive system of behaviour change interventions that acknowledges the capabilities, opportunities, and motivation necessary for smoking cessation. Tsourtos et al. [25] used the Nominal Group Technique to approach a consensus regarding the feasibility and acceptability of six evidence-based resilience interventions among diverse representatives from low SES backgrounds. Setting realistic goals and mindfulness-based interventions were considered the most feasible and appropriate and have therefore been selected for the present study.

Setting goals is one of the most common behaviour change techniques for smoking cessation. [25–27] Goals help regulate behaviour by motivating an individual to reduce the difference between current and desired circumstance. [28] Achieving small goals on the way to tackling more difficult goals can build self-efficacy, confidence, and feelings of agency. [29, 30] Conversely, research suggests that higher quality goals (i.e., clear, proximal quit dates of complete abstinence) can increase the probability of a successful quit attempt. [31] Although specific research on goal setting as a standalone intervention for smoking cessation is scarce, the ability to aid smokers in constructing goals is a critical evidence-based competency for practitioners providing behavioural support. [32] Furthermore, a meta-analysis of 37 intervention studies concluded that goal setting was one of nine techniques which demonstrated the most promising efficacy and feasibility. [27]

There is some evidence that mindfulness-based interventions could be especially effective for low SES smokers. A key reason proposed for the socioeconomic inequity in smoking rates is the chronicity of stressors (including financial strain) and the associated anxiety which disproportionately affects disadvantaged communities. [18, 33–35] Higher trait mindfulness (mindfulness as a stable long-term life approach) has been linked indirectly with a lower risk of smoking relapse through decreased stress levels, indicating that the enhanced emotional regulation afforded by mindfulness might be especially beneficial for low SES smokers trying to quit. [36] Practising mindfulness, as a coping strategy, is also important for low SES smokers because they typically have a higher nicotine dependence. [7, 8] Specifically, mindfulness could decrease perceived withdrawal severity by reducing the agitation and negative affect which accompany nicotine withdrawal and promote resilient responses to cravings. [37, 38] Research also indicates that mindfulness-based addiction therapy might help smokers “bounce back” from lapses and regain abstinence at a higher rate compared to CBT and usual care [39].

Research suggests that peer support programs show promising efficacy for promoting smoking cessation in socioeconomically disadvantaged groups. In their systematic review, Ford et al. [40] argue that this is due to the lower baseline levels of social support often available to disadvantaged groups. Furthermore, a barrier to cessation for disadvantaged groups is that they are sceptical of traditional counselling services (e.g., Quitline), anticipating an impersonal and unsupportive experience. [41] Peer mentors can be perceived as more credible than healthcare professionals because they provide experiential knowledge. [42] By sharing their experiences of quitting and speaking as a peer rather than a professional, peer mentors can be positive role models who might be capable of overcoming perceived impediments to services.

The research described above suggests that that an effective, low-cost approach is necessary to support low SES individuals in quitting smoking and reduce the associated health and economic burdens that affect these communities. Setting realistic goals (SRG) and mindfulness training (MT) are both feasible and potentially effective interventions requiring thorough examination for use with low SES smokers. The present study examines the efficacy of these together with mindfulness-integrated CBT (MiCBT) because MiCBT acknowledges the importance of both as well as CBT.

## Methods

The following protocol (version 5, September 9th 2022) follows SPIRIT guidelines for reporting protocols (see Additional file 1). In this four-arm parallel-group RCT of SRG, MT and MiCBT versus an active control (equal allocation ratio), we will deliver the interventions to small groups of smokers to maximise the potential cost-effectiveness. 6-months of these interventions will be followed by 6-months of online peer support and a further 6-month follow-up period. The control group will be directed to optional resources including counselling (Quitline) and information about pharmacological aid. We aim to: (a) test the efficacy of the three interventions (SRG, MT and MiCBT) for smoking cessation and (b) levels of resilience in low SES groups and how this changes during exposure to the interventions, (c) measure the cost-effectiveness and cost-utility of the interventions in low SES groups, and (d) undertake a process evaluation of the different interventions in low SES groups. We hypothesise the following:

- H1: The proportion of participants who report 14-day period abstinence from smoking at 6 months will be significantly higher for each intervention group compared to the control group.
- H2: Amongst participants that report 14-day period abstinence at 6 months, the proportion of participants who report 14-day period abstinence at 12 months will be significantly higher for each intervention group compared to the control group.
- H3: The proportion of participants who report 14-day period prevalence abstinence from smoking at 6 months post-trial (18 months from baseline) will be significantly higher for each intervention group compared to the control group.
- H4: Levels of resilience measured by the Connor-Davidson CD-25 [43] and ENRICHD Social Support Inventory [44] will be significantly higher for each intervention group compared to the control group at 6, 12 and 18 months post randomisation.
- H5: The interventions will improve smoking cessation and QALYs at a reasonable and acceptable incremental cost.

In addition, we pose the following research questions for the qualitative component of the study (that pertains to the process evaluation):

- RQ1: Have the different interventions been implemented and received as intended?
- RQ2: What are the barriers and facilitators associated with the first research question?
- RQ3: For whom, and in what circumstance were the interventions effective (and cost-effective) or not, and why?

### Participants

We aim to recruit 812 low SES smokers Australia-wide using web-based advertisments. Inclusion/exclusion criteria for this study will require that participants will be aged 18 years or older and have smoked regularly for at least the previous two years, as assessed by the question: “are you a regular smoker (i.e., you have usually smoked at least one cigarette per day for at least the last two years)?” We will only include smokers who are planning to quit, i.e., they answer yes to the question, “are you currently planning to quit smoking cigarettes?” This indicates that the participant is in the preparation stage of the Transtheoretical Model and getting ready to progress to action. [45] Participants must also have a smartphone, have regular internet access, and be willing to spend approximately 14–20 h online to complete the study over an 18-month period.

We define low SES using household income and access to Australian welfare benefits. Smokers will be included if either (a) their weekly household income is below $457 per adult before tax (the Australian poverty line; [46]) or (b) they receive social security benefits, e.g., aged or low-income pension, parenting payments, or disability support payments. Participants will be recruited from all Australian states and territories using online advertisements on social media and news stories on local radio and television. Recruitment will also be promoted via the networks of Project Reference Group members and other stakeholders providing services to low SES populations. Recruitment began on the 3rd of May 2021, and we aim to continue recruitment until the target sample size is reached.

### Sample size and power analysis

For the primary outcome of smoking cessation, 182 participants per group is powered to detect an 11.6% difference [47] in the smoking abstinence rates between groups (with 80% power, alpha = 0.05 (two-tailed test) We will account for a 10% drop-out rate, and thus aim to recruit 203 per group (N = 812). Contextually, the smoking cessation rates we used for the sample size calculation (MT = 25.2%, usual care = 13.6%) represent a meaningful population health treatment effect doubling of the rate of successful quit attempts for the lowest SES group (11.4%; [9]).

### Measures

#### Primary outcome—smoking

The primary outcome is smoking cessation at the 6-month (or 26 week) timepoint. Smoking cessation is defined as 14 days of not smoking cigarettes, determined by two consecutive 7-day periods of self-reported smoking abstinence (“In the last seven days, have you smoked a cigarette, even a puff?”). Participants who meet this criterion at the time of the 6-month mark will be asked to self-administer a saliva test to detect the presence of cotinine (< 30 ng/mL), accurately reflecting abstinence for the preceding 3–4 days. Participants will be instructed to photograph the results and send them to the research team via SMS or email. The purpose of this one-time measurement is to biochemically verify the self-reported smoking cessation outcomes within the study. [48]

#### Secondary outcomes—investigating the mechanisms influencing behaviour change

##### Internal and external resilience

The 25-item Connor-Davidson CD-25 scale measures the internal resilience construct of the psychosocial model of resilience. [43] Connor and Davidson [43] found evidence for the convergent validity of the scale and observed satisfactory internal consistency (α = 0.89) in the general population and satisfactory test–retest reliability (intraclass coefficient = 0.87) in a clinical sample.

The 7-item ENRICHD Social Support Inventory [44] will be used to measure self-reported external resilience. Mitchell [44] found evidence of convergent validity and observed satisfactory internal consistency (⍺ = 0.86). Vaglio et al. [49] observed satisfactory test–retest reliability over a one-month interval (intraclass correlation = 0.94).

##### Self-efficacy

The Smoking Abstinence Self-Efficacy Questionnaire [50] will be used to measure confidence in the ability to change smoking behaviour. Total scores range from 0 to 24, with higher scores indicating greater self-efficacy. Spek et al. [50] observed that the questionnaire had good internal consistency in their sample (α = 0.89) and found evidence for predictive validity.

##### Motivation

Participants’ level of motivation to quit smoking will be measured with Biener and Abrams Contemplation Ladder. [51] This test represents a ladder with ten rungs and five verbal anchors evenly dispersed. Participants select a number from 0 (no thought of quitting) to 10 (taking action to quit). Ladder scores were found to be correlated with the number of previous quit attempts, thereby illustrating construct validity.

##### Stress

The Perceived Stress Scale-10 is a 10-item self-report questionnaire. [52] Lee [53] reviewed the psychometric properties of the scale, finding that all 12 included studies indicated sufficient internal consistency (α > 0.70). Several studies have found evidence for convergent validity. [54, 55]

##### Nicotine dependence

We will assess nicotine dependence with the Fagerstrom Test for Nicotine Dependence, a short, six-item self-report questionnaire. [56] A review of the psychometric properties of the test found excellent test–retest reliability over a range of time periods. [57] Internal consistency was found to be moderate, with ⍺ ranging from 0.55 to 0.74 across 14 studies. Construct validity is evinced by moderate to strong correlations with biological markers of nicotine.

##### Goal achievement

The Problems and Goals Assessment Scale will be administered to SRG participants to measure progress towards goals. [58] The scale consists of a semi-structured evaluation designed to identify what the participant sees as their main problem, its impact, and how it makes them feel. The person rates the degree to which they view this as a problem on a 9-point scale ranging from “not at all” (0) to “a lot” (8). After goals are set to ameliorate the problem, achievement towards the goals is also assessed on a 9-point scale, ranging from “no success” (0) to “complete success” (8). Goal attainment scaling methods are idiosyncratic because they measure an individualised outcome, but their reliability and validity is supported by an extensive literature base. [59]

##### Equanimity

We will use the Equanimity Scale-16 to measure equanimity, which refers to a non-reactive attitude towards experience and is thought to be a key mechanism of action in mindfulness-based interventions. [60] Only participants in the MT and MiCBT arms will be administered the scale. Evidence was found for convergent and divergent validity, and satisfactory internal consistency (⍺ = 0.88) and test–retest reliability (r = 0.87) over a two-to-six-week period was observed. [60]

##### Quality of life

We will measure quality of life with the EQ-5D-5L [61] to assist in estimating the cost-effectiveness of the interventions. The questionnaire comprises a visual analogue scale assessing general health in addition to a 5-component scale including mobility, self-care, usual activities, pain/discomfort, and anxiety/depression. A systematic review found evidence for good reliability and validity across a range of populations. [62] The responses to the 5-component scale questions will be converted to utility scores using an algorithm that reflects Australian population values, noting tariffs for the EQ-5D-5L instrument are in development, but should be available by the time of data analysis. Utility scores are bounded at 1, representing best imaginable health, with a value of zero representing health states that are equivalent to being dead (negative values are possible, representing health states that are determined to be worse than being dead).

#### Costs-effectiveness

Any intervention effects on smoking cessation were not anticipated to lead to differences in health care use (e.g., GP visits, hospitalisations, etc.) between the control and intervention groups over the time horizon of the clinical trial. The lack of an expected effect on health care use did not justify the additional burden of collecting resource use and cost data from participants.

A trial-based economic evaluation will estimate intervention costs, in total, per intervention group participant, per additional intervention group participant demonstrating smoking cessation and per mean change on the EQ5D-5L quality of life instrument. If significant differences in smoking cessation rates are observed, external data describing the long-term health benefits and health care cost impacts will be sought to inform modelled estimates of downstream cost, mortality, and quality of life effects. These data will inform the estimation of the incremental cost per QALY gained.

#### Qualitative data

We will conduct interviews with the counsellors, peer mentors, and participants from all intervention groups to perform a process evaluation. We will focus on barriers and enablers to participation in the study to understand how interventions might effectively translate to a community setting. Questions will be posed regarding feasibility, acceptability, perceived utility, and perception of capabilities, opportunities, and motivations (i.e., components of the COM-B model [24]). Throughout the study, we will also follow up with participants who have missed multiple sessions or have dropped out altogether to record reasons for absence or drop-out. This part of our method will provide a more comprehensive picture of the challenges faced by low SES individuals participating in online smoking interventions. Qualitative analysis will follow a three-phase method: pre-coding; conceptual and thematic categorisation; and theoretical categorisation. [63]

#### Intervention fidelity

We aim to determine intra- and inter-individual variability for intervention session delivery between facilitators. The three facilitators will record sessions of each of the three interventions as they are administered to the first cohort of participants. A member of the research team will review the recordings and apply a bespoke rating system which will assess adherence to the content of the program. Participant engagement and facilitator competence will be gauged during the interview process described above.

### Procedures

**Fig. 2:**
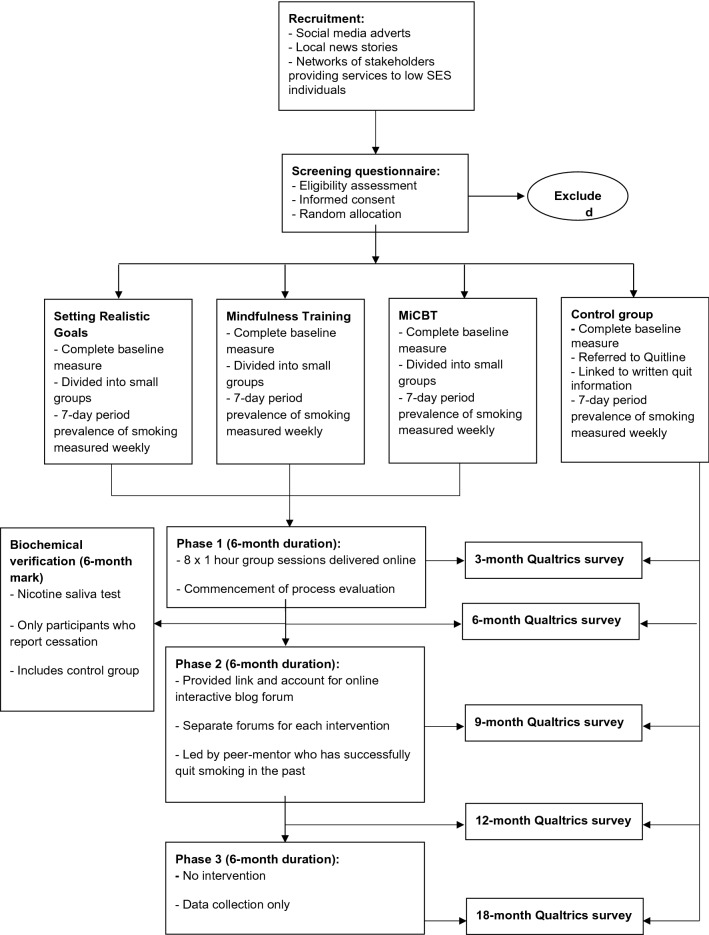
Flow diagram—overview of the procedure

Figure 2 provides an outline of the study design and Table 1 summarises the data collection protocol for secondary outcomes.

**Table 1:**
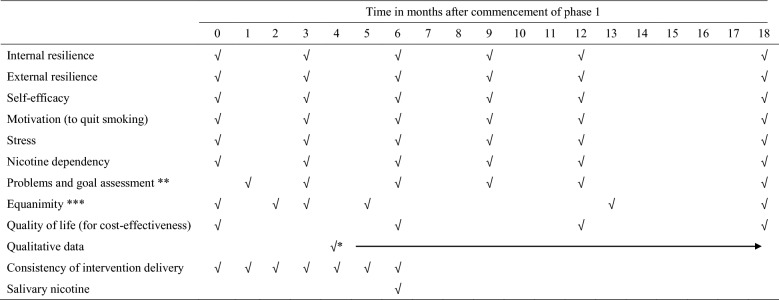
Data collection excluding primary outcome

*Start of qualitative data collection determined by participant drop-out

**Setting realistic goals intervention only; initial assessment occurs in week 5

***Mindfulness-based interventions only; week-based intervals have been approximated into months for clarity

#### Screening and group allocation

Prospective participants will complete an online screening questionnaire that addresses the inclusion criteria through Qualtrics. If eligible, they will be asked to give informed consent. Participants will be randomly allocated to one of the three interventions or a control group using computerised sequence generation within the Qualtrics survey (Mersenne Twister algorithm [64]). All participants will complete a baseline questionnaire (see Table 1). Intervention-group participants will then be divided in groups of between 2 and 7 participants within each of the intervention groups (MiCBT, MT, SRG) based on their availability for session times throughout the week.

#### Phase 1 (months 0–6): group-based interventions

Participants allocated to interventions will attend eight 1-h group sessions online via Zoom over a 6-month period according to a prescribed ideal delivery schedule. Participants will be asked to provide approval these sessions to be recorded (so that they may provide data for analysis regarding intervention delivery, as described above). The facilitators running the interventions will have formal qualifications in CBT and will receive specialised training from experts in mindfulness training and setting realistic goals.

To ensure that all participants are encouraged to access the current best practice approach to smoking cessation, the Royal Australian College of General Practitioner’s [65] “Ask, Advise, Help'” model for smoking cessation will be offered to all participants from baseline, including active control group participants. This approach involves referral to behavioural counselling and provision of information on accessing Nicotine Replacement Therapy and stop-smoking medications where indicated. The behavioural counselling offered in the current study is via the Quitline service.

##### Arm 1

MiCBT is a four-stage therapeutic approach. [66] Therapy begins at the personal stage, with the internal promotion of experiential awareness and acceptance. The second stage introduces imaginary and in vivo exposure procedures, and the third stage extends mindfulness principles into interpersonal interactions. Stage four, the empathetic stage, involves developing ethical awareness and compassion, which is intended to help prevent relapse through counterconditioning. The approach is based on *Vipassana* (insight training) and the co-emergence model of reinforcement. This model proposes that operant behaviour is reinforced by the interoceptive feedback (i.e., conscious or unconscious bodily sensation) which co-emerges with self-referential cognitions, rather than the environmental consequences posited by Skinner. [67] Participants undergoing MiCBT will also be asked to complete 30—60 min of homework daily, including mindfulness practice twice daily and some readings. Homework will be guided by a smartphone application.

##### Arm 2

Participants allocated to the MT arm will learn mindfulness techniques and apply them to change their behaviour. The core concepts will be extracted from the MiCBT intervention, e.g., mindfulness of breath, body-scanning methods, and loving-kindness meditation. However, MT will exclude aspects of CBT which do not directly relate to the meditations. For instance, the first session of MiCBT will focus on attention regulation and weakening peripheral addictions, whereas the equivalent mindfulness training session will only address attention regulation. Participants allocated to this arm will also be asked to perform mindfulness practice twice daily (30–60 min) and access readings through the smartphone application.

##### Arm 3

Participants allocated to arm three will undergo the SRG intervention based on the Problems and Goals Assessment outlined by the Flinders Program. [68] The significant elements of this intervention are problem identification, planning for goal setting, SMART goal setting (specific, measurable, attainable, realistic, time-bound), and reviewing progress. Participants will collaborate to plan goals that may or may not directly pertain to smoking behaviour.

#### Phase 2: peer mentoring

After completing 6 months of group sessions, participants allocated to the three interventions will receive a further 6 months of peer support from a mentor who has achieved smoking cessation in the past. Peers will engage with participants via online blog forums for each intervention. Participants will be encouraged to share their experience with others who underwent the same intervention. These online communities will grow as more users are added upon completion of the group sessions at the 6-month timepoint.

#### Phase 3: data collection only

During the third phase, participants will not receive any intervention from the study, though they might be engaging with other resources such as those provided via the ‘Ask, Advise, Help’ protocol referred to above. We will continue to collect data for a further 6 months after participants have completed the peer support phase. All quantitative data will be collected via online Qualtrics surveys. Links for each Qualtrics survey will be sent to participants by SMS to their mobile phone or by email where the participant requests it.

### Statistical analysis

#### Timepoints

All psychological and behavioural measures (internal resilience, external resilience, self-efficacy, motivation, stress, nicotine dependency, and goal achievement) will be measured before randomisation and then at 3 monthly intervals for the first 12 months (see Table 1; baseline goal assessment at 5 weeks). A final collection of data on these scales will occur at 18 months to test for maintenance of impacts. Equanimity will be measured at baseline, and then weeks 7, 13, 20, 52, and 78 after commencement of phase 1.

In addition to the primary outcome (cessation at 6-months), smoking cessation will be assessed at 4, 8, 12, 16, 20, 52, and 78 weeks to address hypotheses and to compare the trajectory of smoking cessation between groups.

#### Analyses

All analyses will use Stata (version 17.0 [69]). This will be undertaken blinded to the participants’ group allocation. Mixed-effects modelling will be used to assess the effect of the interventions on primary and secondary outcomes. This approach accounts for the correlation of the data from repeat measures over time and reduces bias caused by missing data at one or more time points for each participant. In addition, we will develop prediction models to determine whether levels of internal and external resilience measured at baseline predict smoking cessation. As an exploratory analysis, we will also perform mediation analyses to determine the direct and indirect effects of the separate components of each intervention on the primary and secondary outcomes.

For outcomes assessed at a single time-point, we will assess the difference between the four groups at that time. For outcomes assessed at repeat time points, we will assess differences between the four groups at each time point, with adjustment for multiple comparisons. We will assess binary outcomes using mixed-effects logistic regression and continuous outcomes using linear mixed-effects regression. In repeated measures, we will treat time as a categorical variable, and the model will include fixed-effects for time and group. Subgroup analysis will be undertaken to test whether people with a critical level of resilience are more likely to stay quit than those that do not meet this level. The main analyses will be performed on a per-protocol basis. Our intention-to-treat population will be defined as all participants that were randomised and also completed the baseline survey. We will perform a sensitivity analysis on a modified intention-to-treat basis and impute missing data where possible. Where more than 10% of data are missing, we will also perform multiple imputation as a sensitivity analysis to account for possible bias in estimates caused by missing data. Missing smoking data will be presumed MNAR and analysed as continued smoking. Hypothesis testing will be performed using 2-tailed tests with a Type 1 error rate of alpha = 0.05, and 0.05/3 = 0.0167 when assessing each outcome across time to account for the repeated testing at 6, 12, and 18 months. We will make further adjustments for multiple comparisons (e.g., 3 comparisons with control) depending on each outcome.

#### Cost-effectiveness

A cost-effectiveness model will be used to estimate all important differences in costs and benefits between the three trial arms if there is significant uncertainty regarding the most cost-effective option based on the within trial analysis. Published smoking cost-effectiveness models will be reviewed to identify the most valid available model from an Australian perspective, which will be used to estimate the lifetime incremental costs and QALY gains associated with smoking cessation, as applied in previous economic evaluations of smoking cessation interventions.

### Ethical considerations

There are several ethical considerations associated with this study. To promote an active control group in which the participants receive a usual standard of care, we have elected to expose all participants to the Royal Australian College of General Practitioners Ask, Advise, Help model. Furthermore, both control and SRG group participants will be offered free access to the mindfulness smartphone application after completing the study, should it prove to be of benefit. We also acknowledge that we are studying a vulnerable group. During the ongoing process evaluation, we will “check in” with participants who have dropped out of the interventions to explore how our procedures might be adapted to address any problems they encountered that led to their dropping out and facilitate positive changes in psychological wellbeing. Acknowledging the economic vulnerability of the participant group, we offer intervention group participants who are required to engage with the study via Zoom will be compensated for their data usage with $50 gift cards upon completion of the baseline survey and attendance at their first small group session. For participants who complete the 18-month study, a lottery will be drawn to randomly select winners of supermarket vouchers to the value of $300, $200 and $100. In addition, all control group participants who complete the study will be entered in a lottery to win 1 of 3 x $100 supermarket vouchers to reduce the risk of differential drop-out between study groups. Furthermore, we offer a small reimbursement of $20 to participants who engage with the process evaluation of phase 2 (peer-led blogs).

Study data will be stored on an encrypted drive to maintain confidentiality. The final trial dataset will be accessible by the investigation team. The Project Reference Group members who are external to the research team will function as the Data Monitoring Committee (DMC). The group is comprised on representatives from stakeholder organisations working in tobacco control in addition experts in mindfulness and goal setting interventions. The primary funder will nominate a representative for an observer-only role. The group will provide advice on the project’s feasibility and utility for stakeholder groups considering policy issues, translation of findings, and overall impact on low SES smokers. A formal DMC will not be established due to the minimal risk-levels of the interventions.

### Dissemination

We intend to disseminate the results in peer-reviewed journal articles, press releases, and meetings with stakeholders. Any future amendments this protocol will be found in the relevant trial registry.

### Registration

This study has been registered with the Australian New Zealand Clinical Trials Registry (Trial ID: ACTRN12621000445875). The Universal Trial Number is U1111-1261–8951.

## Discussion

To our knowledge, this study will be the first to compare the efficacy of smoking cessation interventions that are designed to address resilience for low SES smokers, which have been identified by them as acceptable. It will use a randomised controlled trial method in an online delivery format and include peer mentors from the same SES group. The inclusion of psychological and other measures will facilitate exploration of the mechanisms of impact, with a particular focus on the importance of resilience. We expect that the comprehensive picture describing change over time and in response to different interventions, in tandem with the process evaluation, will allow us to determine both efficacy and barriers to translation. If efficacy is established, knowledge of barriers will enable us to further tailor the content and administration of the interventions. By intervening within the framework of the Psychosocial Model of Resilience, we are applying a promising new paradigm to a prominent and inequitable public health problem.

We anticipate several difficulties. Most notably, the interventions will be administered online rather than face-to-face due to social restrictions imposed by the COVID-19 pandemic. This constraint raises questions over the generalisability for face-to-face setting and accessibility to those without ready access to the internet. Nonetheless, testing the efficacy of the interventions in an online setting is important because it reflects real world changes in the way services are provided and supports the inclusion of geographically remote participants. There is evidence that online interventions are effective for addiction treatment (including smoking [70]), and furthermore, there is growing evidence that internet-based CBT can be equivalent to face-to-face CBT in treating a range of psychological problems. [71] Other potential problems raised by the online delivery are lower adherence with intervention protocols and higher drop-out rates. [72] We will try to improve adherence with text message reminders of session times and in-session reminders to complete homework. For the two mindfulness-based interventions, the addition of a recently developed smartphone application provides a simple and user-friendly access route that encourages the completion of homework. We will address the possible attrition bias by using a modified intention-to-treat analysis protocol, whereby participants who drop out of the study will continue to be followed for data collection purposes. We will only request data from participants who have completed the baseline survey.

To summarise, this study aims to test the efficacy of mindfulness and goal-setting interventions with low SES smokers. We will examine the practical application of the interventions to low SES smokers by performing a process evaluation and assessing cost-effectiveness and cost-utility. We expect that our assets-based approach to smoking cessation can guide future public health programs to reduce the prevalence of smoking-related health problems and ameliorate the socioeconomic inequities which continue to be associated with smoking.

## Supplementary Information

**Additional file 1.** SPIRIT Checklist.

**Additional file 2.** Consent Form.

## Data Availability

Deidentified data will be available from though the ANZCTR.

## Abbreviations

DMC: Data monitoring committee
CBT: Cognitive behavioural therapy
MiCBT: Mindfulness-integrated cognitive behavioural therapy
MT: Mindfulness training
QALY: Quality adjusted life year
SACHREC: Southern Adelaide Clinical Human Research Ethics Committee
SES: Socioeconomic status
SMART: Specific, measurable, attainable, realistic, time-bound
SRG: Setting realistic goal
