# Supplementary material for: Efficacy of mindfulness and goal setting interventions for increasing resilience and reducing smoking in lower socio-economic groups: randomised controlled trial protocol
Source: Addict Sci Clin Pract. 2023 Feb 6;18:7. doi: 10.1186/s13722-022-00355-w (PMC9900553; doi:10.1186/s13722-022-00355-w)
Supplement: Supplementary file 2 — Additional file 2. Consent Form. [file 13722_2022_355_MOESM2_ESM.docx]

**INFORMATION SHEET**

**Title:** Increasing resilience and reducing smoking for lower socio-economic groups.

**Project number:** 270.19

**Researcher(s)**

College of Medicine and Public Health, Flinders University: Dr George Tsourtos ([tsou0021@flinders.edu.au](mailto:tsou0021@flinders.edu.au)), Prof Paul Ward ([paul.ward@flinders.edu.au](mailto:paul.ward@flinders.edu.au)), Dr. Emma Miller (emma.miller@flinders.edu.au), Prof Sharon Lawn ([sharon.lawn@flinders.edu.au), Pro](mailto:sharon.lawn@flinders.edu.au),%20Pro)f Carlene Wilson (carlene.[wilson@flinders.edu.au](mailto:wilson@flinders.edu.au)), Prof Richard Woodman ([richard.woodman@flinders.edu.au](mailto:richard.woodman@flinders.edu.au)), Prof Jonathan Karnon (jonathan.karnon@flinders.edu.au),

Ms Elissa Mortimer (elissa.mortimer@flinders.edu.au).

**Introduction**

You are invited to take part in this research project. This Information Sheet tells you about the research project. Knowing what is involved will help you decide if you want to take part.

Please read this information carefully. Before deciding whether or not to take part, you might want to talk about it with a relative, friend or local doctor. Participation in this research is voluntary.

If you decide you want to take part, you will be asked to complete the consent section by ticking ‘Agree’ at the end. This tells us that you:

- understand what you have read
- consent to take part
- consent to the use of your personal information as described.

You can download a copy of this Information Sheet to keep here.

**Purpose of the study**

This project will test ways to increase resilience to help people who want to quit smoking. Increasing resilience has been identified by smokers as a potentially useful way to improve quit success.

**What will I be asked to do?**

You will be randomly put into one of 4 study groups. All of these groups will participate in the study for 18 months in total. Depending on which group you are in, you may:

- participate in 8 online group sessions over the first 6-month period with a counsellor who is trained in strategies for quitting smoking. You will then be connected to an online forum for a further 6 months with other people who are participating in this study and peer mentors who have successfully quit smoking; and/or
- be provided with a referral to the Quitline service that provides behavioural counselling about quitting cigarettes by phone plus written information to help you to quit and access nicotine replacement therapy if you choose;

During the study, if you quit smoking, you may be asked to do a saliva test to check your cotinine levels. Cotinine is a by-product of nicotine which is present in tobacco. A saliva testing kit will be mailed out to you by post at no charge to you.

Over the total 18 months of the study, you will be asked to complete surveys online every few months. This should take about 20-30 minutes each time. If you prefer, we can send you these surveys by post with a reply-paid envelope.

**What benefit will I gain from being involved in this study?**

The first potential benefit is that you may successfully quit smoking. Secondly, at the end of the study, all participants will be provided with free access to an app which may provide further help to quit or stay quit. Overall, there may be no direct benefit to you from participating in this study, but you will help us work out how to support current and future smokers on their quitting journey.

**Additional costs & reimbursement**

If you are in a group that requires you to participate in online group sessions, you will be provided with a $50 supermarket voucher to offset the cost of your data usage.

Participants will also go into a draw to win a Coles shopping voucher. Three vouchers will be awarded to the value of $100, $200 and $300.

**Will I be identifiable by being involved in this study?**

The research team will need to know your contact details and first name during the course of the study. All identifying information will be removed at the end of the study. All information and results obtained in this study will be stored in a secure way, with access restricted to relevant researchers. It is possible that other study participants will know your name if you are in one of the counselling groups. The counsellor will advise group members that information shared should not be discussed outside the consultation sessions.

You will be asked at the beginning of these group sessions if you agree to the session being audio recorded. If you do not agree, it will not affect your participation or the group session. The session will still be held and not audio recorded. If you and the other members of your group do agree, the audio recording will be used by the Flinders study team to find out how similar or different the delivery of your group was to other groups.

In any publication and/or presentation about this study, you will not be identified, except with your permission. No individual results or any contact details for participants will be reported.

**Are there any risks or discomforts if I am involved?**

Some participants may experience emotional discomfort such as withdrawal symptoms when attempting to quit smoking. Below are free services that you may want to access if you require support/counselling:

- Quitline 13 78 48
- Beyond Blue 1300 22 4636
- Lifeline 131 114

**How do I agree to participate?**

To agree to participate, please read the acknowledgement comments below. If you agree, click ‘ACCEPT’ and then you will be taken to the first batch of surveys for the study.

**Complaints and compensation**

If you suffer any injuries or complications as a result of this research project, you should contact the study team as soon as possible.

**How will I receive feedback?**

You can request a copy of the findings of the study at any time from the research team via email.

**Who has reviewed the research project?**

This research project has been approved by the Southern Adelaide Clinical Human Ethics Research Committee (Project number 270.19). For queries regarding the ethics approval of this project please contact the Southern Adelaide Clinical Executive Officer on 82046453 or email at Health.SALHNOfficeforResearch@sa.gov.au. If you have any complaints or concerns please contact the Director of Research Operations via telephone on 82046453 or email Health.SALHNOfficeforResearch@sa.gov.au.

**Acknowledgment**

1. I have read the information provided.
2. Details of procedures and any risks have been explained to my satisfaction.

3. I am aware that I should retain a copy of the Information Sheet and Consent Form for future reference.

4. I understand that:

- I may not directly benefit from taking part in this research.
- Participation is entirely voluntary and I am free to withdraw from the project at any time; and am free to decline to answer any questions.
- While the information gained in this study will be published as explained, my participation will not be anonymous; however, any information I provide will remain confidential.
- Whether I participate or not, or withdraw after participating, will have no effect on any treatment or service that is being provided to me.

5. I understand that only the researchers on this project will have access to my research data and raw results; unless I explicitly provide consent for it to be shared with other parties. If the need to seek consent to share research data with other parties does arise, I will be contacted by the researchers

**□ ACCEPT**
